# Causality relationship between 91 inflammatory factors and Alzheimer disease: A bidirectional Mendelian randomization study

**DOI:** 10.1097/MD.0000000000048136

**Published:** 2026-04-24

**Authors:** Qijia Li, Shunyou Jing, Ning Li, Xiaokui Yuan, Tong Wang

**Affiliations:** a Department of Clinical Laboratory, Sichuan Provincial Women’s and Children’s Hospital/The Affiliated Women’s and Children’s Hospital of Chengdu Medical College, Chengdu, China; b Department of Clinical Laboratory, The Fourth People’s Hospital of Chengdu/The Clinical Hospital of Chengdu Brain Science Institute, University of Electronic Science and Technology of China, Chengdu, China.

**Keywords:** Alzheimer disease, causality relationship, inflammatory factors, Mendelian randomization

## Abstract

Alzheimer disease (AD) is a neurodegenerative disorder characterized by amyloid plaque deposition, neurofibrillary tangles, and chronic neuroinflammation. Due to its complexity and difficult-to-treat nature, it has cast a huge shadow over global health. In addition to genetic susceptibility, the development of AD is closely related to systemic inflammation. This study aims to evaluate the association between systemic inflammatory factors and AD through a bidirectional Mendelian randomization (MR) design. Our MR design incorporated aggregated data from extensive genome-wide association studies to investigate the causal relationship between genetically determined systemic inflammatory factors and AD. The MR analysis results identified 9 potential systemic inflammatory regulatory factors: C-X-C motif chemokine 5, interleukin-18 receptor 1, interleukin-6, and tumor necrosis factor, which were associated with an increased risk. Conversely, AD is significantly correlated with 5 circulating inflammatory regulatory factors, namely, tumor necrosis factor-related apoptosis-inducing ligand, stem cell factor, monocyte chemoattractant protein-4, interleukin-5, and cystatin D, which are considered downstream consequences of AD. It is worth noting that our results have, for the first time, clarified the significant roles of inflammatory factors such as cystatin D and monocyte chemoattractant protein-4 in AD, providing new markers and key targets for further exploration of the molecular mechanism and clinical diagnosis and treatment of AD.

## 1. Introduction

Alzheimer disease (AD) is a neurodegenerative disorder characterized by progressive cognitive dysfunction, with hallmark pathological features including aberrant β-amyloid (Aβ) deposition, hyperphosphorylated tau protein, neurofibrillary tangles, neuronal loss, and synaptic damage.^[1–3]^ Emerging evidence suggests that, beyond these core pathological alterations, neuroinflammation plays a pivotal role in AD pathogenesis. However, the existing observational studies investigating the association between inflammatory mediators and AD often fail to adequately account for potential confounding factors (e.g., comorbid conditions, pharmacotherapy), nor can they definitively discern whether alterations in inflammatory markers represent a causative factor or a consequence of AD. Consequently, elucidating the causal relationship between inflammatory cytokines and AD risk holds significant scientific implications for unraveling disease mechanisms and developing novel therapeutic strategies.

Over the past few decades, despite substantial investment in targeted therapeutic research for AD, progress has remained limited. This therapeutic impasse largely stems from an incomplete understanding of the intricate pathophysiological mechanisms underlying AD.^[4]^ Notably, circulating inflammatory mediators – key regulators of the immune microenvironment – have emerged as a focal point in research exploring their association with AD pathological progression.^[5,6]^ In recent years, large-scale genome-wide association studies (GWAS) have identified multiple single-nucleotide polymorphisms (SNPs) significantly associated with AD risk, among which autosomal dominant mutations in the amyloid precursor protein, presenilin 1 (*PSEN1*), and presenilin 2 (*PSEN2*) genes have been conclusively linked to the pathogenesis of familial AD.^[7,8]^ However, conventional observational studies exhibit notable limitations: they often fail to fully account for potential confounding factors (e.g., unmeasured environmental exposures) and lack the capacity to definitively establish temporal causality. Consequently, there is an urgent need to employ more robust analytical approaches to investigate the causal relationship between alterations in inflammatory mediator levels and the onset of AD.

MR analysis represents an innovative methodological approach that employs genetic variants as instrumental variables (IVs), providing a robust framework for elucidating causal relationships between metabolic factors and disease outcomes while effectively circumventing the reverse causation and confounding biases that frequently undermine observational studies.^[9,10]^ This unique advantage establishes MR as the gold-standard analytical tool for investigating exposure-outcome causality in epidemiological research.

In this study, we employed an extensive 2-sample MR framework to investigate the causal relationship between genetically predetermined circulating inflammatory factors and the risk of AD. By leveraging summary statistics derived from large-scale GWAS encompassing inflammatory regulators and AD susceptibility, we identified 9 inflammatory factors exhibiting significant causal associations with AD. Our findings not only highlight potential biomarkers and therapeutic targets for the early diagnosis and precision treatment of AD but also provide novel mechanistic insights into the pathogenesis of this disease.

## 2. Materials and methods

### 2.1. MR design and data source

**Figure 1.**
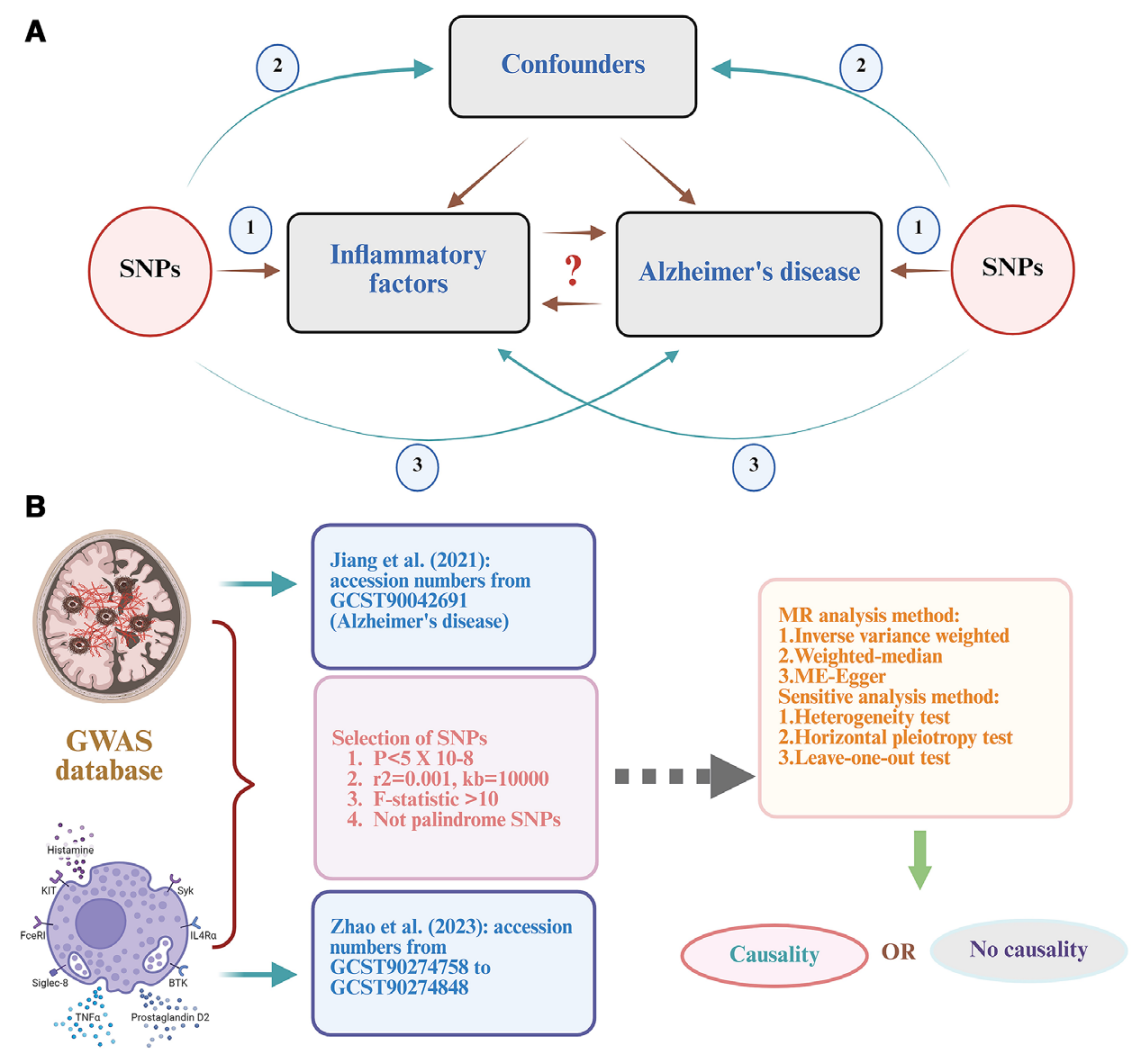
(A) Three assumptions in this MR study: diagram of MR principles and assumptions. Associational assumption: the IVs must be robustly associated with the exposure of interest. Exclusion restriction assumption: the IVs influence the outcome solely through the exposure. Independence assumption: the IVs are independent of any confounding factors. (B) Workflow of this MR study. IV = instrumental variable, MR = Mendelian randomization, SNPs = single-nucleotide polymorphisms.

This study employed a bidirectional 2-sample Mendelian randomization (MR) approach, using genetic instruments (SNPs) from the latest GWAS to predict systemic inflammatory factors and AD. We utilized a bidirectional MR design to assess the association between systemic inflammatory factors and AD, and to test whether AD causes systemic inflammatory factors. The general design of this MR research is stated in Figure 1. Genetic association data for systemic inflammatory regulators and AD were obtained from the GWAS catalog (https://www.ebi.ac.uk/gwas/downloads/summary) or the IEU GWAS database (https://gwas.mrcieu.ac.uk/datasets/). After strict quality control, 91 inflammatory factors were analyzed from 14,824 participants of European ancestry (accession numbers from GCST90274758 to GCST90274848).^[11,12]^ The datasets of AD were obtained from the GWAS Catalog (Dataset: GCST90042691), comprising a total of 356,042 participants, including 2087 cases and 353,955 controls.^[13]^ All data were derived from published studies or publicly available GWAS abstract data in which ethical approval and informed consent were provided. No separate ethical approval was required for this study.

### 2.2. Selection of IVs

First, SNP loci extracted from the AD genome-wide association study summary data must achieve genome-wide significance (*P* value < 5 × 10^−8^). Second, there should be no linkage disequilibrium (LD) between any SNPs. To assess the LD relationships among SNPs, a clustering analysis was performed using sample data from the 1000 Genomes Project (with parameters set to *R*^2^ = 0.001 and a window size of 10,000 kb), and SNP loci missing from the LD reference panel were excluded. Finally, SNP loci with a minor allele frequency below 0.01 were removed.^[14]^ The aforementioned SNP loci were extracted from the outcome GWAS data. After excluding missing SNPs, the alleles for the exposure factors and outcome effects were matched and calibrated. The SNPs that passed this stringent filtering process were then used as IVs for subsequent 2-sample MR analyses. According to the fundamental assumptions of MR analysis, SNPs used as IVs must be strongly associated with the exposure factors. To evaluate the strength of these IVs, we calculated the *F*-statistic for each SNP. The *F*-value >10 indicates that the likelihood of bias due to weak instruments is minimal. The *F*-statistic was calculated by the following equation: *F* = *R*^2^ × (N − *k* − 1)/*k* × (1 − *R*^2^), where N is the sample size of the exposure factor, *k* is the number of IVs, and *R*^2^ is the proportion of variance explained by each IV.^[15]^

### 2.3. Statistical analyses

This study employed a 2-sample MR approach to investigate the causal effects of inflammatory factors on AD. The causal associations between inflammatory factors and AD were estimated using the following 3 methods: inverse-variance weighted (IVW) method under a random-effects model,^[16]^ weighted median estimator, and MR-Egger regression.^[17]^ The random-effects IVW method was the most commonly used approach in MR analyses and provides robust causal estimates in the absence of directional pleiotropy.^[18]^ The MR-Egger method can test for violations of the IV assumptions, but it had lower estimation precision and was susceptible to the influence of outlier genetic variants.^[19]^ The weighted median method effectively mitigates interference from invalid IVs and can yield consistent causal effect estimates when more than 50% of the weight comes from valid SNPs.^[20]^ Compared with the MR-Egger method, this approach reduced type I error rates and enhanced the accuracy of causal estimates. Among these, the IVW method provided the most precise estimates but was susceptible to horizontal pleiotropy and outliers.^[9]^ Therefore, it was chosen as the primary method for this analysis. Results were presented as odds ratios (OR) with corresponding 95% confidence intervals (CI). The IVW method calculated the causal estimate by meta-analyzing the Wald ratios of each SNP (i.e., the β coefficient of the outcome SNP divided by the β coefficient of the exposure SNP).

We also employed the MR-Egger and MR-PRESSO methods to detect horizontal pleiotropy. When heterogeneity or horizontal pleiotropy was identified, we conducted sensitivity analyses by reanalyzing the data after excluding outlier SNPs identified by the MR-PRESSO method. The *P* value >.05 indicated no evidence of pleiotropy. In cases where these methods yielded inconsistent results, we prioritized the conclusions from the IVW method as the primary basis for our judgment.

We employed the Cochran *Q* test to assess heterogeneity among SNPs. A *P* value >.05 indicated no significant heterogeneity. In addition, a leave-one-out analysis was conducted to examine the impact of individual SNPs on the causal relationship between exposure and outcome. The statistical analysis revealed no evidence of horizontal pleiotropy (*P* > .05). To account for multiple testing, this study applied the Bonferroni correction. Associations with *P* values below .0012 (0.05/41) were considered strong evidence, while those with *P* values between .0012 and .05 were regarded as suggestive evidence.^[21]^

All statistical analyses and results visualization utilized R statistical software (version 4.4.2, https://www.R-project.org) with the “Two Sample MR,” “forestplot,” and “MRPRESSO” packages.

## 3. Result

### 3.1. Characteristics of IVs and exposure factors

Following the application of predefined screening criteria, SNPs meeting the thresholds were extracted from the GWAS database and employed as IVs (see Supplementary Material, Tables S1–S10, Supplemental Digital Content, https://links.lww.com/MD/R711, for comprehensive details). Baseline characteristics of the 91 circulating inflammatory regulators are summarized in Table S1, Supplemental Digital Content, https://links.lww.com/MD/R711. Owing to the extensive number of inflammatory mediators and their corresponding SNPs, this section predominantly highlights statistically significant causal associations derived from the MR analyses.

The SNPs utilized in both forward and reverse MR analyses have been systematically catalogued in Tables S1 to S10, Supplemental Digital Content, https://links.lww.com/MD/R711, encompassing genomic coordinates, effect alleles, allele frequencies, and pertinent functional annotations. Notably, all selected SNPs exhibited *F*-statistics exceeding the threshold of 10, thereby confirming the robustness of the IVs and effectively mitigating potential weak instrument bias.

### 3.2. Pleiotropy and heterogeneity of genetic IVs for AD and inflammatory factors

**Figure 2.**
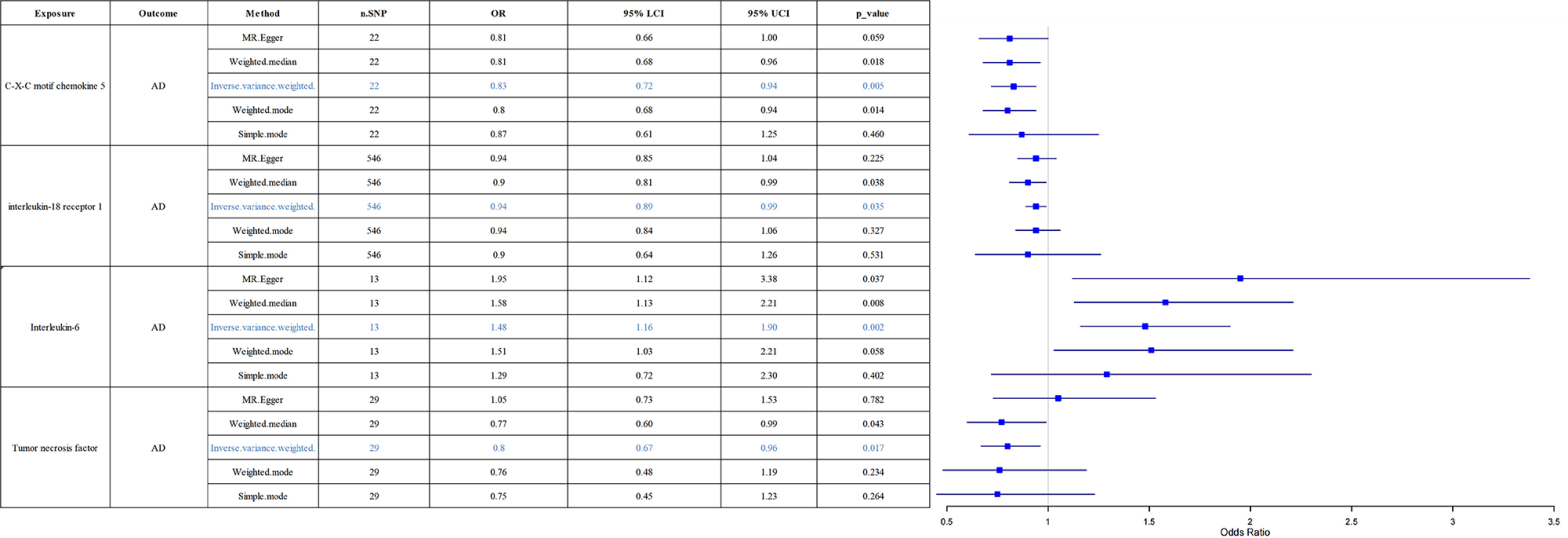
Forest plots of the pooled OR results between 4 inflammatory factors and AD in the forward MR analysis. OR (95% CI) means risk of AD per 1 SD increase of continuous factors or per 1 unit log odds increase of binary factors. The primary results of the MR analyses were derived from the inverse-variance weighted method, with these findings clearly indicated in the results section. AD = Alzheimer disease, CI = confidence interval, LCI = lower confidence interval, MR = Mendelian randomization, OR = odds ratio, SD = standard deviation, SNPs = single-nucleotide polymorphisms, UCI = upper confidence interval.

**Figure 3.**
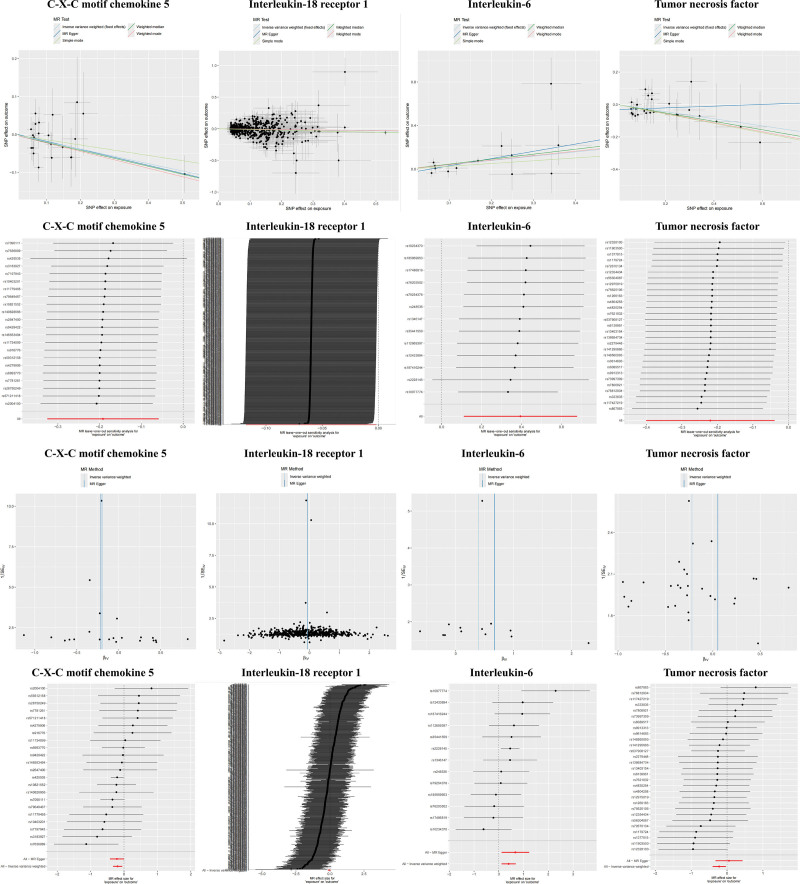
MR analysis. Scatter plot, leave-one-out plot, funnel plot, and forest plot of inflammation factors to AD. AD = Alzheimer disease, MR = Mendelian randomization, SNPs = single-nucleotide polymorphisms.

In the present study, we employed MR analyses to elucidate the genetic interplay between AD and inflammatory factors. Based on the results of IVW analyses, a total of 9 inflammatory factors were identified to exhibit putative causal associations with AD. Although the IVW approach is highly effective in inferring causal relationships between exposures and disease outcomes, it is known to be susceptible to weak instrument bias. To rigorously assess the robustness of these putative causal associations, we conducted comprehensive sensitivity analyses. The sensitivity analysis ultimately corroborated the presence of potential bidirectional causal relationships between AD and these 9 inflammatory factors (Figs. 2 and 3). Notably, these 9 inflammatory factors demonstrated substantial consistency across a range of MR analytical methods. For the assessment of heterogeneity, we employed both MR-Egger regression and the IVW approach to calculate Cochran *Q* statistics; the aggregate results indicated the absence of detectable heterogeneity (Table 1). Taken together, the MR analyses exhibited no evidence of horizontal pleiotropy (*P* > .05) or heterogeneity (*P* > .05; Table 1). All statistical tests were conducted using two-sided procedures, with the significance threshold set at α = 0.05. Analytical methodologies strictly adhered to international standards to ensure the reliability of the findings.

**Table 1:** The table of heterogeneity test and pleiotropy test of MR analysis.

Exposure	Outcome	Heterogeneity test			Pleiotropy test		
		MR-Egger	IVW		IVW		
		*Q*-value	*Q*-df	*P* value	Intercept	SE	*P* value
C-X-C motif chemokine 5	AD	0.654	21	.709	0.003	0.014	.809
Interleukin-18 receptor 1	AD	0.076	545	.081	0.000	0.004	.957
Interleukin-6	AD	0.212	12	.190	0.037	0.033	.285
TNF	AD	0.912	28	.850	0.031	0.019	.114

Outcome	Exposure	Heterogeneity test			Pleiotropy test		
		MR-Egger	IVW		IVW		
		*Q*-value	*Q*-df	*P* value	Intercept	SE	*P* value
TNF-related apoptosis-inducing ligand	AD	0.370	30	.418	0.002	0.008	.823
Stem cell factor	AD	0.776	30	.664	0.014	0.008	.084
Monocyte chemoattractant protein-4	AD	0.187	30	.223	0.000	0.009	.971
Interleukin-5	AD	0.470	30	.512	0.004	0.009	.643
Cystatin D	AD	0.887	30	.742	0.016	0.008	.044

AD = Alzheimer disease, IVW = inverse-variance weighted, MR = Mendelian randomization, SE = standard error, TNF = tumor necrosis factor.

### 3.3. Causal impact of inflammatory factors on AD

When considering 91 circulating inflammatory factors as exposures and the functional outcome of AD as the endpoint, the IVW analysis revealed that elevated levels of C-X-C motif chemokine 5 (CXCL5, OR = 0.83, 95% CI = 0.72–0.94, *P* = .005), interleukin-18 receptor 1 (IL-18R1, OR = 0.94, 95% CI = 0.89–0.99, *P* = .035), and tumor necrosis factor (TNF, OR = 0.8, 95% CI = 0.67–0.96, *P* = .017) were inversely correlated with the risk of AD. Conversely, higher levels of interleukin-6 (IL-6, OR = 1.48, 95% CI = 1.16–1.90, *P* = .002) were positively associated with an increased incidence of AD (Fig. 2). Figure 3 presents the results of IVW, weighted median, MR-Egger, and simple mode analyses, all of which consistently demonstrate significant causal relationships between AD and these 4 inflammatory factors.

### 3.4. The causal impact of AD on systemic inflammatory factors

**Figure 4.**
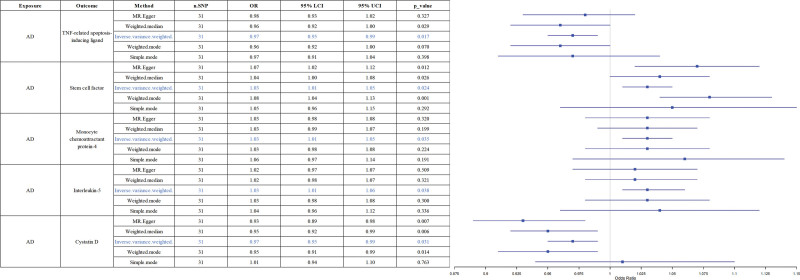
Forest plots of the pooled OR results between 5 inflammatory factors and AD in the reverse MR analysis. OR (95% CI) means level of inflammatory factor per 1 SD increase of continuous factors or per 1 unit log odds increase of binary factors. The primary results of the MR analyses were derived from the inverse-variance weighted method, with these findings clearly indicated in the results section. CI = confidence interval, LCI = lower confidence interval, MR = Mendelian randomization, OR = odds ratio, SD = standard deviation, SNPs = single-nucleotide polymorphisms, UCI = upper confidence interval.

**Figure 5.**
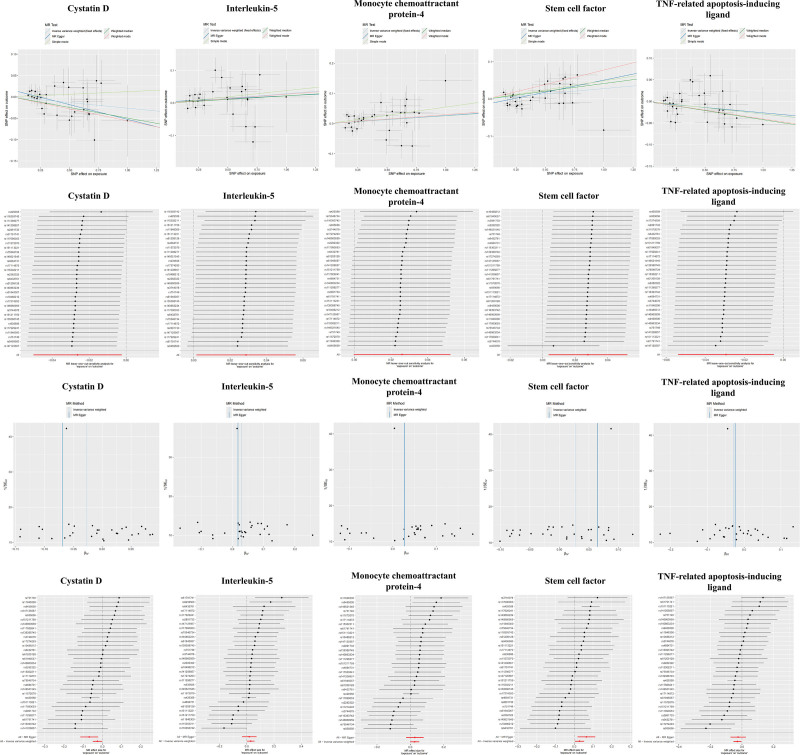
MR analysis. Scatter plot, leave-one-out plot, funnel plot, and forest plot of AD to inflammation factors. AD = Alzheimer disease, MR = Mendelian randomization, SNPs = single-nucleotide polymorphisms, TNF = tumor necrosis factor.

When AD was designated as the exposure and 91 circulating inflammatory factors were considered as functional outcomes for reverse analysis, the results of the IVW approach indicated that AD exhibited a positive association with stem cell factor (OR = 1.03, 95% CI = 1.01–1.05, *P* = .024), monocyte chemoattractant protein-4 (OR = 1.03, 95% CI = 1.01–1.05, *P* = .035), and interleukin-5 (IL-5, OR = 1.03, 95% CI = 1.01–1.06, *P* = .038). In contrast, AD was negatively correlated with cystatin D (OR = 0.97, 95% CI = 0.95–0.99, *P* = .031) and TNF-related apoptosis-inducing ligand (OR = 0.97, 95% CI = 0.95–0.99, *P* = .017). These biomarkers are purported to represent downstream secondary effects of AD (for details of the analysis, see Fig. 4). Figure 5 depicts the results of IVW, weighted median, MR-Egger, and simple mode analyses, collectively demonstrating the presence of significant causal relationships between AD and these 4 inflammatory factors.

## 4. Discussion

Recent advances have increasingly highlighted the pivotal role of immune dysregulation and neuroinflammation in the pathogenesis of AD.^[22]^ Among various inflammatory mediators, key cytokines and chemokines have been demonstrated to contribute to disease progression and offer potential avenues for therapeutic intervention.^[23]^

Within the TNF superfamily, TNF-α is a prototypical pro-inflammatory cytokine extensively studied in the context of AD. Preclinical models have shown that both prophylactic and therapeutic inhibition of TNF-α substantially alleviates neuropathological abnormalities and enhances cognitive function. Clinical trials further support the therapeutic promise of TNF-α inhibitors, which can slow cognitive decline and improve daily functioning in patients with AD.^[24–26]^ Collectively, these findings underscore the significance of TNF-α as a molecular nexus mediating the “crosstalk” between peripheral immune responses and central nervous system pathology, highlighting anti-TNF-α strategies as promising immunomodulatory therapies for neurodegenerative conditions.^[27]^ IL-6 is a multifunctional cytokine implicated in immunity and inflammation. Elevated IL-6 expression is closely linked to AD pathology, associated with increased Aβ burden and patterns of cerebral atrophy, including hippocampal and cortical loss, as observed in both clinical and preclinical studies.^[28]^ Experimental evidence, including our own data, supports the capacity of IL-6 to induce neuronal injury and impair cognitive function, further substantiating its pathogenic relevance to AD.^[29,30]^ The cystatin gene family, particularly cystatin C, has attracted attention due to its inhibitory effects on Aβ aggregation and plaque formation; however, the involvement of other family members in AD has remained largely unexplored.^[31,32]^ Our genetic findings identify a potential association between cystatin D (CST5) and AD risk, suggesting novel roles for the cystatin superfamily in neurodegenerative processes. Chemokines have also emerged as mediators of neuroinflammation in AD. Monocyte chemoattractant protein-4/CC-chemokine ligand 13 (CCL13), which orchestrates monocyte and eosinophil recruitment, has previously been understudied in relation to AD.^[33]^ In contrast, CCL2, a related chemokine, has been linked to disease progression and cognitive decline.^[34]^ Our findings, for the first time, suggest a genetic involvement of CCL13 in AD, raising the possibility that it may serve as a biomarker for disease progression and represent a novel therapeutic target. Furthermore, TRAIL (tumor necrosis factor-related apoptosis-inducing ligand) has garnered notice for its ability to induce apoptosis in malignant cells. Accumulating evidence suggests that TRAIL is upregulated in AD and contributes to neuronal apoptosis and neuroinflammation.^[35,36]^ Intriguingly, our study highlights a significant reduction of TRAIL expression with AD progression, an observation aligning with the complex and context-dependent roles of this molecule in neurodegeneration. Beyond these extensively studied factors, we provide the first genetic evidence implicating CXCL5, IL-18R1, SCF (stem cell factor), and IL-5 in AD pathogenesis. Although the precise functions of these mediators in AD remain to be clarified, their identification in our analyses offers new directions for future mechanistic studies and the development of targeted therapies.

In summary, our study expands the repertoire of inflammatory mediators implicated in AD, not only corroborating the involvement of established cytokines such as TNF-α and IL-6, but also unveiling novel candidates, including CST5, CCL13, CXCL5, IL-18R1, SCF, and IL-5. These findings advance our understanding of the complex inflammatory networks driving neurodegeneration and may inform innovative strategies for AD intervention and biomarker discovery. Despite these significant findings, several limitations should be acknowledged. First, while the MR approach employed in this study provides statistical evidence supporting a causal relationship between circulating inflammatory factors and AD, the precise biological mechanisms underlying these associations remain to be clarified in future experimental investigations. Furthermore, as the genetic data analyzed were derived primarily from individuals of European ancestry, the extent to which these results can be generalized to other ethnic groups or geographic populations is uncertain. Subsequent studies are warranted to replicate these findings in more diverse cohorts and to further elucidate the mechanistic pathways by which inflammatory mediators contribute to AD pathogenesis, thereby improving the robustness and applicability of our conclusions.

## 5. Conclusion

This study employed a bidirectional MR approach to systematically elucidate the causal relationships between inflammatory mediators and AD. The results demonstrate that fluctuations in the levels of inflammatory factors such as CXCL5, IL-18R1, and TNF may act as driving forces in the pathogenesis of AD, functioning as upstream regulatory elements. In contrast, elevated IL-6 is likely to directly facilitate the onset and progression of AD. On the contrary, alterations in the concentrations of SCF, monocyte chemoattractant protein-4, IL-5, CST5, and TRAIL appear more plausibly to represent secondary consequences – downstream regulatory factors – of the pathological process of AD.

The inflammatory mediators identified herein may thus serve as promising biomarkers for the early diagnosis of AD while simultaneously furnishing novel perspectives for comprehending the pathophysiological mechanisms underlying AD. Moreover, the causal network delineated by this research provides a theoretical framework for multiple potential molecular targets for precision intervention in AD. Future investigations will focus on elucidating the specific molecular mechanisms through which these key inflammatory mediators contribute to the initiation and progression of AD, and on exploring targeted therapeutic strategies anchored in these discoveries. Such efforts are anticipated to open up innovative avenues for the prevention and treatment of AD.

## Author contributions

**Data curation:** Qijia Li, Tong Wang.

**Formal analysis:** Qijia Li.

**Funding acquisition:** Qijia Li, Ning Li.

**Investigation:** Shunyou Jing, Xiaokui Yuan, Tong Wang.

**Methodology:** Shunyou Jing, Tong Wang.

**Validation:** Xiaokui Yuan.

**Visualization:** Xiaokui Yuan.

**Project administration:** Tong Wang.

**Resources:** Tong Wang.

**Software:** Tong Wang.

## Supplementary Material
